# On the use of thermal forces to probe kinesin’s response to force

**DOI:** 10.3389/fmolb.2023.1260914

**Published:** 2023-10-31

**Authors:** Chuan Chang, Tiantian Zheng, Guilherme Nettesheim, Hayoung Song, Changhyun Cho, Samuele Crespi, George Shubeita

**Affiliations:** ^1^ Physics Program, New York University Abu Dhabi, Abu Dhabi, United Arab Emirates; ^2^ Cavendish Laboratory, Department of Physics, University of Cambridge, Cambridge, United Kingdom

**Keywords:** molecular motors, kinesin, intracellular transport, motor force response, motor runlength, thermal forces

## Abstract

The stepping dynamics of cytoskeletal motor proteins determines the dynamics of cargo transport. In its native cellular environment, a molecular motor is subject to forces from several sources including thermal forces and forces ensuing from the interaction with other motors bound to the same cargo. Understanding how the individual motors respond to these forces can allow us to predict how they move their cargo when part of a team. Here, using simulation, we show that details of how the kinesin motor responds to small assisting forces–which, at the moment, are not experimentally constrained-can lead to significant changes in cargo dynamics. Using different models of the force-dependent detachment probability of the kinesin motor leads to different predictions on the run-length of the cargo they carry. These differences emerge from the thermal forces acting on the cargo and transmitted to the motor through the motor tail that tethers the motor head to the microtubule. We show that these differences appear for cargo carried by individual motors or motor teams, and use our findings to propose the use of thermal forces as a probe of kinesin’s response to force in this otherwise inaccessible force regime.

## 1 Introduction

Long distance transport within eukaryotic cells is crucial for cell maintenance and survival. Cargo, vesicles and organelles, are carried by molecular motors which step along the actin and microtubule cytoskeletal filaments, with the latter being responsible for long range transport. Along microtubules, which have distinct ends, the dynein motor walks toward the minus-end, while motors in the kinesin family are responsible for transport in the plus-end direction. Kinesin-1 (henceforth referred to as kinesin) has been extensively studied *in vitro* and at the single-molecule level, providing precise measurements of many of its properties. Kinesin is processive and moves in 8.2 nm steps, commensurate with the underlying microtubule structure (Howard et al., 1989; Svoboda et al., 1993). Kinesin hydrolyzes ATP to power its stepping, and at saturating ATP concentrations it moves at speeds of around 1 μm/s, and slows down with hindering forces up to a stall at around 5 pN (Svoboda and Block, 1994; Block, 2007). Kinesin’s mechanochemical cycle leads to a finite probability of detachment from the microtubule at each step resulting in an average run-length of around one hundred steps (∼900 nm). The motor’s detachment probability is dependent on the magnitude and direction of the force it is subjected to, leading to an exponential decrease of run-length with hindering force and a precipitous drop in run-length with assisting forces as small as 2 pN (Milic et al., 2014; Andreasson et al., 2015). The response of the motor to assisting forces below 2 pN has not been reported.

In cells, teams of kinesin motors bind to and move individual cargos (Welte et al., 1998; Shubeita et al., 2008; Hendricks et al., 2012; Leidel et al., 2012; Rai et al., 2013). Experimental efforts to understand the dynamics of motor teams *in vivo* and, with increasing levels of complexity, *in vitro* have unraveled important questions relevant to cellular transport (Gross et al., 2002; Mallik et al., 2005; Martinez et al., 2007; Vershinin et al., 2007; Shubeita et al., 2008; Derr et al., 2012; Hendricks et al., 2012; Jamison et al., 2012; Leidel et al., 2012; Xu et al., 2012; Blehm et al., 2013; Rai et al., 2013; Arpag et al., 2014; Bergman et al., 2018; Arpag et al., 2019; Tjioe et al., 2019; Li et al., 2023). However, given the limitation of what is experimentally feasible, simulations and models have also contributed to understanding cargo dynamics in various geometries and under various conditions (Kunwar et al., 2008; Korn et al., 2009; Kunwar and Mogilner, 2010; Kunwar et al., 2011; Arpag et al., 2014; McLaughlin et al., 2016; Arpag et al., 2019; Jiang et al., 2019; Khataee and Howard, 2019; Ohashi et al., 2019; Wilson et al., 2019; Bovyn et al., 2021; Yadav and Kunwar, 2022). Simulating cargo transport has been possible thanks to the great level of detail with which the biochemical and mechanochemical properties of single kinesin motors have been measured. However, as with every complex system, the outcome of the simulation can depend considerably on the details of the model. The ensuing variability is not just an *in silico* artifact, as attempts to mimic the cellular transport by multiple motors *in vitro* have produced diverse outcomes determined by what motor variant is used, the cargo geometry, and how the motors are linked to the cargoes.

With kinesin being a mechanoenzyme whose enzymatic cycle is modulated by external forces, thermal forces acting on the cargo and forces developing as members of a cargo-bound motor team interact can result in emergent responses of these motors. These forces are transduced to the motors through their long tail domains and neck linkers and, therefore, the stiffness of these domains that tether the microtubule-bound motor head to the cargo can play an important role in determining cargo dynamics. In this work, we use simulation to study the effect of the tether stiffness on the dynamics of cargo driven by single kinesin motors. We find that changing the details of the force response of the motor in the force regime not yet accessible to experiment can produce distinct cargo dynamics even in this seemingly simple system. These effects carry over in systems of multiple motors. With the different models leading to distinct dynamics under the influence of thermal forces alone, we propose a mechanism for using thermal forces to experimentally constrain models of kinesin’s response to force.

## 2 Results

### 2.1 The run-length of a single motor increases with tether stiffness

We have designed a stochastic simulation of a single kinesin-1 hauling a 0.5 µm cargo, incorporating the known properties of the motor (see Methods for details). The response of the kinesin motor to assisting and hindering forces has been mapped out with great detail underscoring its asymmetric response to forces in the opposing directions. Kinesin-1 slows down when hindered, but its speed does not change when subject to an assisting force (Andreasson et al., 2015). Similarly, its run-length, the average distance it travels before falling off the microtubule, drops gradually with increasing hindering force but drops precipitously with a slight assisting force (Milic et al., 2014). We therefore modeled the run-length as two piecewise exponential drops as a function of force.

**FIGURE 1:**
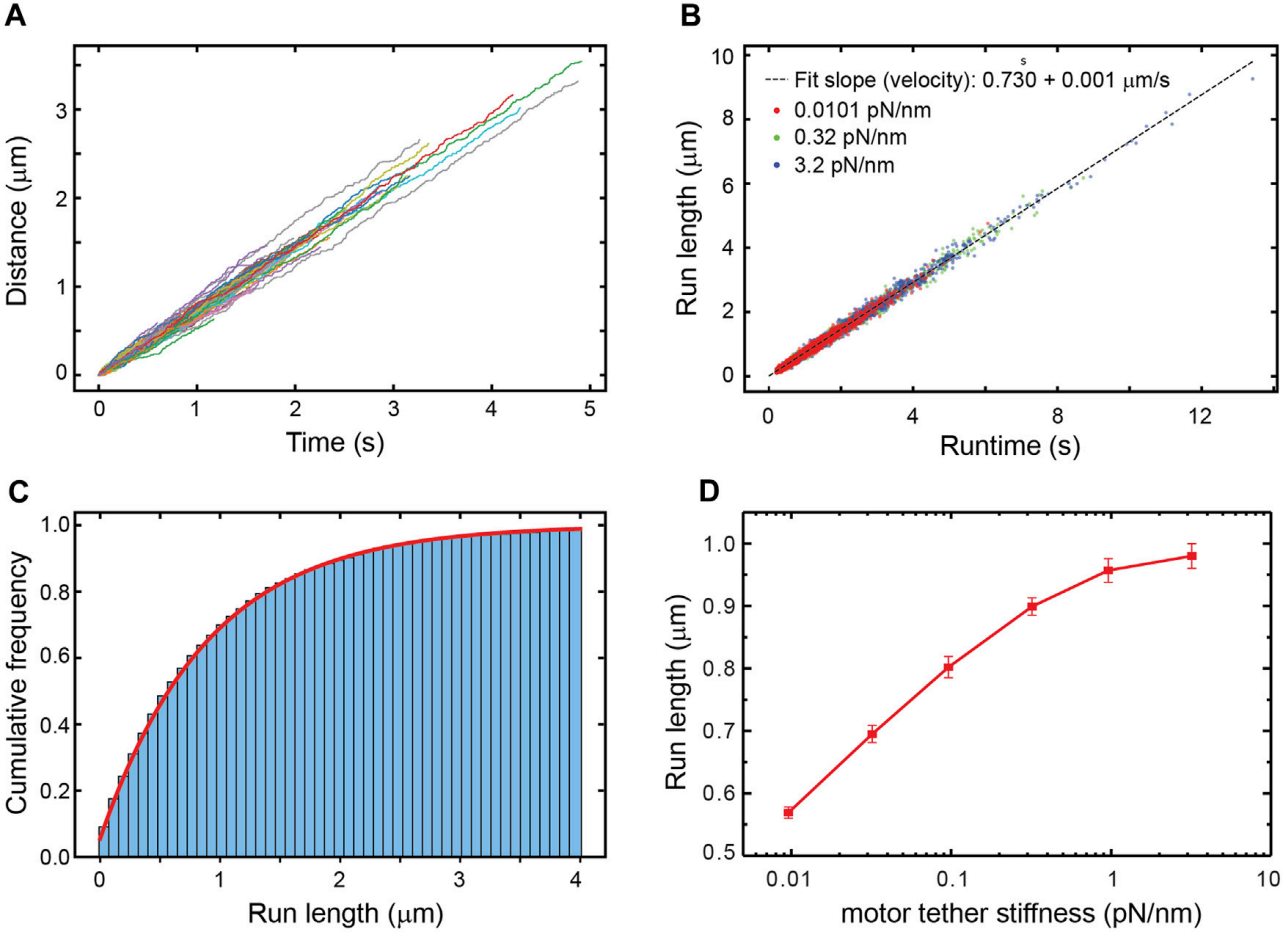
**(A)** A random sample of 105 simulated trajectories of beads carried by a single kinesin-1 motor. The simulation accounts for thermal forces and Stokes’ drag on the bead but no additional external forces are applied. **(B)** The run-length *versus* runtime for trajectories simulated with different stiffnesses of the kinesin-1 motor tether that links it to the bead. The distribution of velocities and its mean value are not altered by the tether stiffness. **(C)** Cumulative distribution of run-lengths of bead trajectories simulated using the measured value of kinesin’s tether stiffness (0.32 pN/nm). The fit to the underlying exponential distribution gives an average run-length of 870 nm. **(D)** The average run-length vs. tether stiffness.

The simulation corresponds to unloaded bead motility assays where no external force is applied. The trajectories produced by the simulation reproduce the expected distribution of velocities and run-lengths (Figures 1A–C). Because the kinesin tether, which is comprised of the tail and neck linker, mechanically couples the cargo to the biochemically-active kinesin head, we surmised that its stiffness might have an effect on the motor’s run-length. We therefore varied the stiffness from 3% of its reported value in full-length kinesin (Jeney et al., 2004) to 10 times its reported value. Surprisingly, we find that the run-length increases by around 400 nm over this range of stiffness (Figure 1D).

### 2.2 The sensitivity of the run-length to tether stiffness depends on the details of motor detachment probability

**FIGURE 2:**
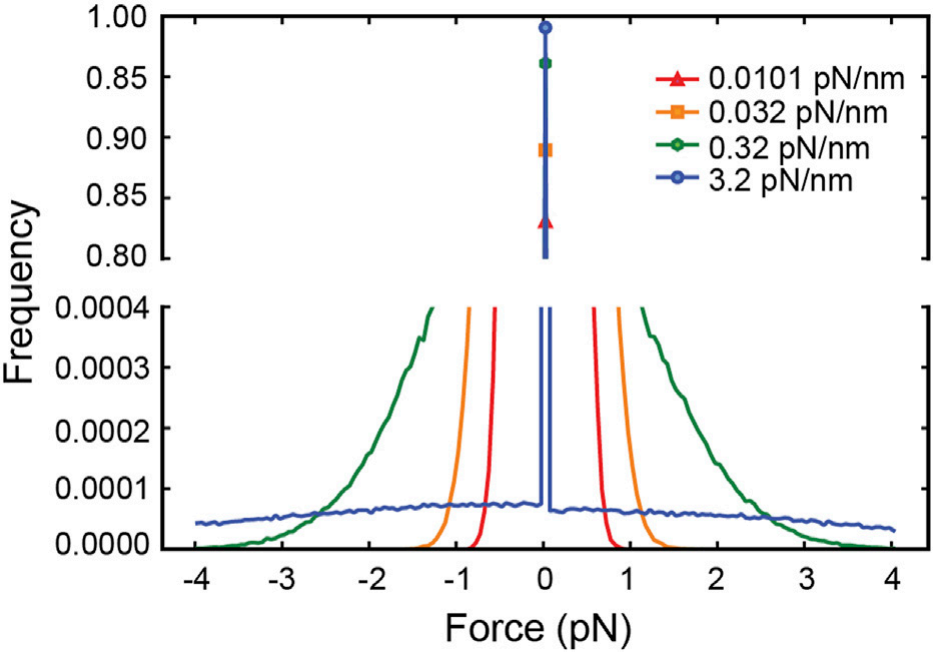
The distribution of net forces experienced by the simulated kinesin-1 motor due to thermal forces on the cargo for different tether stiffnesses. Since the cargo diffuses freely until the tether is stretched, the motor experiences zero force most of the time. As the tether stiffness increases, the frequency of non-zero force decreases even if it is larger on average.

Since external forces are the only factor that can affect a motor’s run-length in the simulation, the increase in run-length with the kinesin tether stiffness (Figure 1D) implies that the average force experienced by the motor changes with the stiffness. Viscous drag forces acting on the cargo, which are in turn transmitted to the motor through the elastic tether, cannot be responsible for this dependence of run-length on stiffness since the velocity distribution is the same for all tether stiffnesses (Figure 1B). Thermal forces jiggling the cargo around are the only other source of force and act randomly, resulting in both hindering and assisting forces. With thermal forces acting on the cargo and stretching the tether, a stiffer tether transmits a larger force when stretched, yet a limper tether has a larger probability of transmitting a force larger than zero (Wilson et al., 2019) (Figure 2), resulting in more frequent motor detachment and a smaller average run-length, as seen in Figure 1.

**FIGURE 3:**
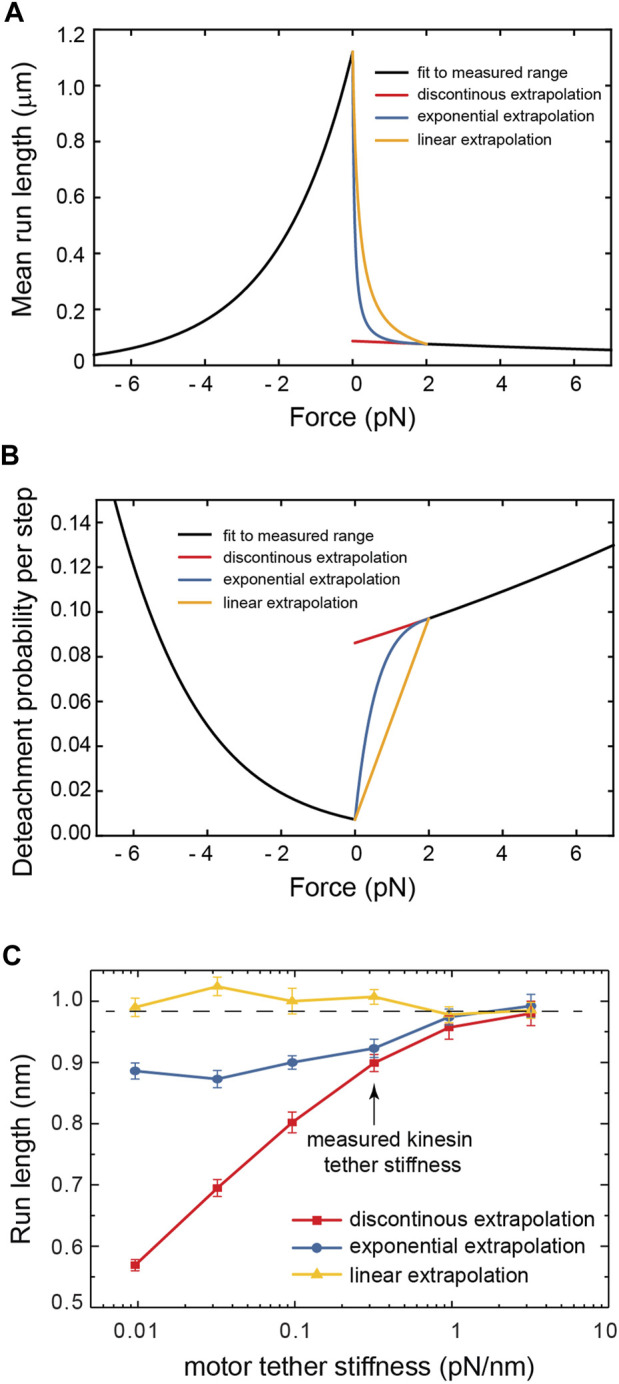
**(A)** The three models of the kinesin-1 force-dependence of the run length used in this work. **(B)** The detachment probability per step corresponding to the run length responses in **(A)**. **(C)** The run length of cargo carried by motors of varying tether stiffness for the three detachment probability models.

The distribution of forces in Figure 2 shows that for tether stiffnesses smaller than 0.32 pN/nm, the forces experienced by the motor are predominantly smaller than 2 pN. This suggests that for such small net forces the shape of kinesin’s response matters. However, assisting forces smaller than 2 pN are precisely those not accessible to experiment (Milic et al., 2014)! To cover this experimental gap, we extrapolated the exponential fit of the run-length response to assisting forces as reported by Milic et al. This results in a discontinuity in the response at zero force (Figures 3A,B), which is unlikely a good model of kinesin’s real response. Previously, Wilson *et al.* bridged the force response by linearly extrapolating kinesin’s detachment rate in the force range inaccessible to experiment (Wilson et al., 2019). This resolves the discontinuity at zero force and, therefore, it is a better model of kinesin’s response (Figures 3A,B). They showed that the linear extrapolation resulted in a run-length response that is insensitive to tether stiffness, and it was explained by the near-perfect compensation of the increased load exerted by stiffer tethers, which tend to shorten run-length, and the reduced likelihood of stiffer tethers to exert a non-zero force. As shown in Figure 2C, implementing this model in our simulation results in a similar flat response, despite some differences in the details of the simulation implementation (see methods). This linear extrapolation, however, is still unlikely a good model of kinesin’s response since it is not smooth at 2 pN and, importantly, may also underestimate the detachment probability of the motor in this small assisting force regime. Given the marked difference in run-length response between the discontinuous and linear extrapolations, we implemented a smoother exponential extrapolation of the motor detachment probability between 0 pN and the first reported measurement at 2 pN (Figures 3A,B). This detachment probability resulted in a weak but significant increase in the run-length with tether stiffness (Figure 3C). Over the range of stiffnesses explored, the run-length increased by around 100 nm.

### 2.3 The run-length, but not the velocity, of cargo carried by two motors is sensitive to the detachment model

**FIGURE 4:**
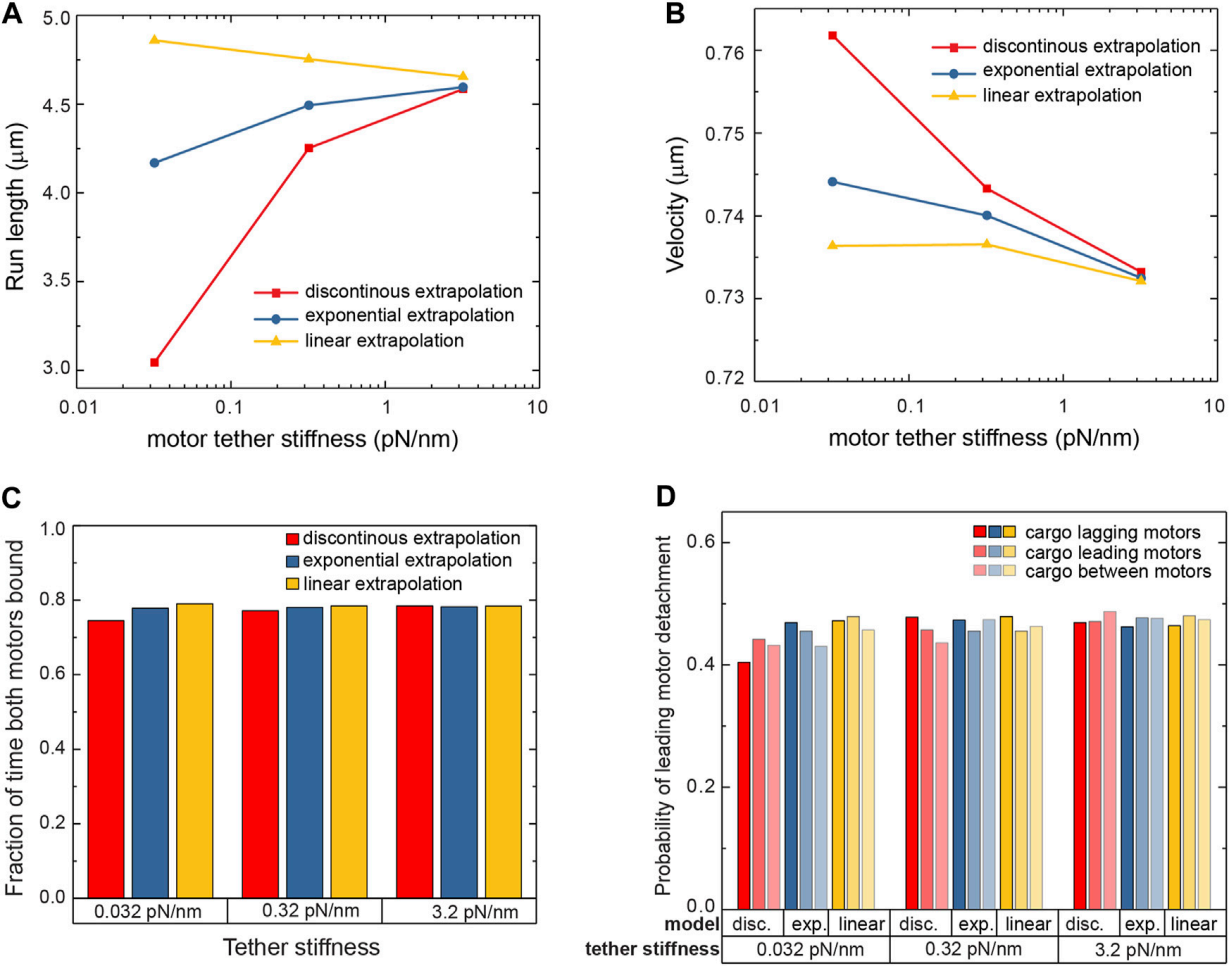
The run-length **(A)** and velocity **(B)** of cargo carried by two kinesin-1 motors are presented for various tether stiffnesses and for the three detachment probability models. While the run-length is spread over a wide range, the velocity shows variation smaller than 5% over that wide range of parameters and models used in the simulation. **(C)** Two motors are bound to the microtubule during the run most of the time, for all stiffnesses and all models. **(D)** Small variations are observed in the likelihood that the leading motor of the team detaches.

We next investigated the response of cargoes carried by two kinesin-1 motors. Multiple motors bind to the same cargo in living cells (Shubeita et al., 2008; Hendricks et al., 2012; Leidel et al., 2012; Shubeita and Gross, 2012; Blehm et al., 2013; Rai et al., 2013), and when part of a team, the stochastic stepping of each motor results in the distance between the motors fluctuating. There will therefore be instances when the motors exert forces on each other, with the leading and lagging motors experiencing hindering and assisting loads, respectively (Nettesheim et al., 2020). These loads will act in addition to thermal forces, which were alone sufficient to reveal the differences in the individual motor sensitivity to force. We simulated a system of two motors attached to the bead using the three aforementioned detachment models and a range of tether stiffnesses. Each simulation terminated when both motors were detached from the microtubule at the same time, ending the run. The run-length of these trajectories can be significantly longer than that of single motor trajectories since when one motor detaches the other still holds the bead close to the microtubule allowing the detached motor to rebind (Nettesheim et al., 2020; Wilson et al., 2021). We find that both motors are bound to the microtubule simultaneously more than 74% of the time for all models and all tether stiffnesses (Figure 4C). Nevertheless, the run-length sensitivity to the tether stiffness is different for the different detachment models used (Figure 4A). Similar to the single motor, the discontinuous model results in a large sensitivity of the run-length on the tether stiffness. The linear extrapolation model and the exponential extrapolation model are less sensitive, with around a 5% change in run-length over two orders of magnitude of stiffness.

With the two motors exerting forces on each other, and given the directional responses of the motor’s run-length and velocity to force, the velocity of a cargo driven by two motors could depend on the detachment model used and the tether stiffness. Changes in velocity could come about in multiple ways. First, instances when the motors are far apart and experience forces in opposite directions can result in the leading motor slowing down while the lagging motor’s velocity remains unchanged (Andreasson et al., 2015). Second, the different detachment probabilities depending on the direction of the force (Figure 3B) can lead to more frequent detachments of the lagging motor. Elastic and thermal forces would then lead the cargo to advance to the position of the bound leading motor, resulting, on average, in an increase in its velocity. Drag and thermal forces additionally modulate the forces acting on the motors and therefore their velocities and detachment frequencies. However, we find that the velocity of the cargoes driven by two motors changes by less than 5% across the various detachment models and over two orders of magnitude of tether stiffness (Figure 4B). Nevertheless, the general trend is a small decrease in the velocity with increasing stiffness.

## 3 Discussion

We found that the details of kinesin’s detachment probability at small assisting forces, currently inaccessible to experiment, influence cargo run-length significantly. Reconciling the different models used to simulate transport with experimental findings using different kinesin constructs and different geometries implemented to mimic cargo transport *in vitro*, requires a detailed understanding of the individual motor’s properties. Our finding that, for some models, the run-length of the cargo can be influenced by the tether’s stiffness suggests that care must be taken in interpreting experimental findings as the stiffness of the kinesin tether can be affected by its length and by any linker used to attach the motor to the cargo. Conversely, one can use the predictions of the simulation to constrain models of the actual load-detachment probability of the motor by means of *in vitro* experiments where the tether stiffness is varied using engineered linkers. The thermal forces experienced by the motors lie precisely within the force range presently inaccessible to experiment (Figure 2). Our finding that thermal forces alone are sufficient to differentiate between the load-detachment models suggests that such an experiment would be quite feasible. It is further intriguing that these differences persist even for transport by teams of motors where the run-length is much longer, dictated by the rate of rebinding to the microtubule after a motor detaches (Wilson et al., 2021), suggesting that multiple motor assays could also be informative. Moreover, since kinesin-1 velocity has been shown to follow an Arrhenius temperature dependence over an extended range (Hong et al., 2016), motility assays performed at various temperatures could be used to further constrain the models.

For very stiff motor tethers, the run-length converges to the same value for all detachment models. Under these conditions, the motor rarely experiences forces larger than zero (Figure 2) and, therefore, the details of how it detaches under load become less relevant. The fact that the run-length of cargo carried by two stiff motors is independent of the detachment model is also not surprising. For the stiffest tethers simulated (3.2 pN/nm), when the motors step far apart so that the distance between them is twice their relaxed length, any additional step of the leading motor increases the force by around 13 pN, beyond the force regime that differentiates the models. It is worth noting that much of the dynamics of cargo transport is of course guided by the coupling of the force to the motor. The fact that kinesin is stiff in extension but not in compression (Jeney et al., 2004) allows the cargo to diffuse over a large range without transmitting any appreciable force to the motor. Similarly, the motors can separate by a large distance before they start feeling tension. The dynamics would drastically change if the kinesin tether was significantly shorter. It is intriguing to speculate that the change in cargo dynamics with motor tether length and stiffness may be one adaptation that results in different kinesin family members having different tail lengths.

Unlike run-length, the velocity of the cargo is largely insensitive to the details of the detachment probability at small assisting forces. This is not surprising for cargo carried by a single motor since detachment simply ends the run. However, for two motors, this finding is not trivial. Every time one of the motors detaches, the motor-cargo complex centers around the remaining motor (Nettesheim et al., 2020). This results in a forward or backward shift for lagging or leading motor detachment, respectively, so that a bias in the detachment probability would translate into a speedup or slowdown. Additionally, hindering force slows down the motor whereas assisting force does not. The two motors experience force either because they step apart or due to thermal forces on the cargo. The former is only relevant for smaller tether stiffnesses when the force generated per step beyond the relaxed tether length is below 2 pN. In this regime, the lagging motor is more likely to detach causing a cargo speedup that depends on the exact detachment model. However, since tension between the motors slows down the leading motor but not the lagging one, these instances do not persist for long. The thermal forces, by swaying the cargo, can cause an additional difference in the likelihood of the lagging and leading motor detachment, depending on the relative position of the cargo and motors as well as the separation between the motors (Figure 3D). While the different models we simulated do result in distinct velocity responses to the tether stiffness, the differences are small and more so for stiffer tethers. Nevertheless, it is intriguing that the complex dynamics of differential detachment probabilities and force-velocity responses results in just small changes in cargo velocity. On the other hand, that the unloaded velocity is relatively insensitive to the stiffness of the motor tether even for cargoes carrying more than one motor, implies that any manipulation that leads to changing cargo velocity can be very informative (Nettesheim et al., 2020).

## 4 Methods

Our code modeled the 1-D travel of a spherical cargo (0.5 µm in diameter) carried by one or two kinesin motors along a microtubule at 25°C. Motors stochastically transitioned through states representing the kinesin stepping cycle, and they were coupled to the cargo and to each other through the forces they exerted on the cargo. The motion of the cargo was modeled using overdamped Langevin dynamics (Kunwar et al., 2008). The velocity of the cargo is caused by the combination of the force exerted by the motors and the external delta-correlated random force used to represent the thermal noise. The simulation corresponds to unloaded bead motility assays where no additional external force is applied. Each motor behaves as an elastic band which exerts a Hookian force upon extension beyond a natural length L = 50 nm, but not upon compression (Jeney et al., 2004). The cargo diffuses freely as long as the motor is unstretched, and experiences tethered diffusion beyond kinesin’s natural length. At the beginning of the simulation, the cargo was placed at position 0, and the head end of all motors were attached to the microtubule with positions uniformly distributed on the interval [−L, L] (i.e., no motors were stretched). While the two heads of kinesin were not explicitly simulated, kinesin was allowed to be in the three attached substates, and one detached state, corresponding to processes involving the two heads within one step (Andreasson et al., 2015). Substate 1 transitioned into substate 2 with rate per second $k_{1}\left(F\right)=k_{1}^{0}\exp\left[F\delta_{1}/k_{B}T\right]$ , where F is the force acting on the motor and $\delta_{1}$ is the characteristic distance parameter for this transition; substate 2 transitioned into substate 3 with constant rate per second $k_{2}$ ; substate 3 transitioned into substate 1 with rate per second $k_{3}\left(F\right)=k_{3}^{0}\exp\left[\frac{\left(F+F_{T}\right)\delta_{3}}{k_{B}T}\right]$ , where *F*_*T*_ is the inter-head tension (Andreasson et al., 2015); substate 2 transitioned into the off-state with probability per step given by $p_{\det}\left(F\right)=\frac{8.2}{L_{p}}\exp\left[\frac{\left|F\right|\delta_{p}}{k_{B}T}\right]$ for the discontinuous model at all values of *F*, and for the linear and exponential models for F≤0pN and F≥2pN . In the small assisting force range ( 0<F≤2pN ) the detachment probability is given by $p_{\det}\left(F\right)=0.00727+0.0449F$ for the linear extrapolation model, and $p_{\det}\left(F\right)=0.1-\left[0.1-p_{det}\left(0\right)\right]\times \exp\left[\frac{1}{2}\ln\left(\frac{0.1-p_{\det}\left(2pN\right)}{0.1-p_{\det}\left(0pN\right)}\right)\times F\right]$ for the exponential extrapolation model, where $p_{\det}\left(0\right)$ and $p_{\det}\left(2pN\right)$ are the measured detachment probabilities at 0 and 2pN, respectively. The off-state transitioned into the on-state with a rate of 5 s^−1^ (Leduc et al., 2004). The values for these parameters are shown in Table 1. At each time step, the force F by the cargo on the motor was calculated (this is equal to the Hookian force exerted by the motor on the cargo if the motor was stretched beyond length L), and, from this the probability of transitioning to the next (sub)state was calculated. If a motor transitioned from substate 2 to substate 3, it had made a step, and the position of the motor was increased by 8.2 nm. If a motor transitioned from substate 2 to the off state, it had detached from the microtubule and the run would end if the motor was the only motor or the last attached motor. Otherwise, the detached motor could re-attach in the next time steps at a position uniformly distributed on the interval [X_c_ − L, X_c_ + L], where X_c_ is the position of the cargo in that timestep. The probabilities of transitioning for each motor were independent from those of the other motor in the two-motor simulations.

**TABLE 1:** Simulation parameters.

Parameter	Value Andreasson et al. (2015)
$k_{1}^{0}$	$4900s^{-}1$
$\delta_{1}$	4.6 nm
$k_{2}$	$95s^{-}1$
$k_{3}^{0}$	$260s^{-}1$
$\delta_{3}$	0.35 nm
$F_{T}$	26 pN
$L_{p}$	$L_{p}=L_{H}=1120nm,F\leq 0$
	$L_{p}=L_{A}=87nm,F>0$
$\delta_{p}$	$\delta_{p}=\delta_{H}=2.0nm,F\leq 0$
	$\delta_{p}=\delta_{A}=0.27nm,F>0$

## Data Availability

The original contributions presented in the study are included in the article/supplementary material, further inquiries can be directed to the corresponding author.

## References

[B1] Andreasson J. O. L. Milic B. Chen G. Y. Guydosh N. R. Hancock W. O. Block S. M. (2015). Examining kinesin processivity within a general gating framework. Elife 4, e07403. 10.7554/eLife.07403 25902401 PMC4453223

[B2] Arpag G. Norris S. R. Mousavi S. I. Soppina V. Verhey K. J. Hancock W. O. (2019). Motor dynamics underlying cargo transport by pairs of kinesin-1 and kinesin-3 motors. Biophys. J. 116, 1115–1126. 10.1016/j.bpj.2019.01.036 30824116 PMC6428962

[B3] Arpag G. Shastry S. Hancock W. O. Tuzel E. (2014). Transport by populations of fast and slow kinesins uncovers novel family-dependent motor characteristics important for *in vivo* function. Biophys. J. 107, 1896–1904. 10.1016/j.bpj.2014.09.009 25418170 PMC4213720

[B4] Bergman J. P. Bovyn M. J. Doval F. F. Sharma A. Gudheti M. V. Gross S. P. (2018). Cargo navigation across 3D microtubule intersections. Proc. Natl. Acad. Sci. U. S. A. 115, 537–542. 10.1073/pnas.1707936115 29295928 PMC5776960

[B5] Blehm B. H. Schroer T. A. Trybus K. M. Chemla Y. R. Selvin P. R. (2013). *In vivo* optical trapping indicates kinesin's stall force is reduced by dynein during intracellular transport. Proc. Natl. Acad. Sci. U. S. A. 110, 3381–3386. 10.1073/pnas.1219961110 23404705 PMC3587256

[B6] Block S. M. (2007). Kinesin motor mechanics: binding, stepping, tracking, gating, and limping. Biophys. J. 92, 2986–2995. 10.1529/biophysj.106.100677 17325011 PMC1852353

[B7] Bovyn M. Janakaloti Narayanareddy B. R. Gross S. Allard J. (2021). Diffusion of kinesin motors on cargo can enhance binding and run lengths during intracellular transport. Mol. Biol. Cell 32, 984–994. 10.1091/mbc.E20-10-0658 33439674 PMC8108528

[B8] Derr N. D. Goodman B. S. Jungmann R. Leschziner A. E. Shih W. M. Reck-Peterson S. L. (2012). Tug-of-War in motor protein ensembles revealed with a programmable DNA origami scaffold. Science 338, 662–665. 10.1126/science.1226734 23065903 PMC3840815

[B9] Gross S. P. Welte M. A. Block S. M. Wieschaus E. F. (2002). Coordination of opposite-polarity microtubule motors. J. Cell Biol. 156, 715–724. 10.1083/jcb.200109047 11854311 PMC2174082

[B10] Hendricks A. G. Holzbaur E. L. Goldman Y. E. (2012). Force measurements on cargoes in living cells reveal collective dynamics of microtubule motors. Proc. Natl. Acad. Sci. U. S. A. 109, 18447–18452. 10.1073/pnas.1215462109 23091040 PMC3494964

[B11] Hong W. Takshak A. Osunbayo O. Kunwar A. Vershinin M. (2016). The effect of temperature on microtubule-based transport by cytoplasmic dynein and kinesin-1 motors. Biophys. J. 111, 1287–1294. 10.1016/j.bpj.2016.08.006 27653487 PMC5034348

[B12] Howard J. Hudspeth A. J. Vale R. D. (1989). Movement of microtubules by single kinesin molecules. Nature 342, 154–158. 10.1038/342154a0 2530455

[B13] Jamison D. K. Driver J. W. Diehl M. R. (2012). Cooperative responses of multiple kinesins to variable and constant loads. J. Biol. Chem. 287, 3357–3365. 10.1074/jbc.M111.296582 22158622 PMC3270990

[B14] Jeney S. Stelzer E. H. K. Grubmuller H. Florin E. L. (2004). Mechanical properties of single motor molecules studied by three-dimensional thermal force probing in optical tweezers. Chemphyschem 5, 1150–1158. 10.1002/cphc.200301027 15446737

[B15] Jiang R. Vandal S. Park S. Majd S. Tuzel E. Hancock W. O. (2019). Microtubule binding kinetics of membrane-bound kinesin-1 predicts high motor copy numbers on intracellular cargo. Proc. Natl. Acad. Sci. U. S. A. 116, 26564–26570. 10.1073/pnas.1916204116 31822619 PMC6936695

[B16] Khataee H. Howard J. (2019). Force generated by two kinesin motors depends on the load direction and intermolecular coupling. Phys. Rev. Lett. 122, 188101. 10.1103/PhysRevLett.122.188101 31144901 PMC12175961

[B17] Korn C. B. Klumpp S. Lipowsky R. Schwarz U. S. (2009). Stochastic simulations of cargo transport by processive molecular motors. J. Chem. Phys. 131, 245107. 10.1063/1.3279305 20059119

[B18] Kunwar A. Mogilner A. (2010). Robust transport by multiple motors with nonlinear force-velocity relations and stochastic load sharing. Phys. Biol. 7, 16012. 10.1088/1478-3975/7/1/016012 20147778 PMC2858005

[B19] Kunwar A. Tripathy S. K. Xu J. Mattson M. K. Anand P. Sigua R. (2011). Mechanical stochastic tug-of-war models cannot explain bidirectional lipid-droplet transport. Proc. Natl. Acad. Sci. U. S. A. 108, 18960–18965. 10.1073/pnas.1107841108 22084076 PMC3223464

[B20] Kunwar A. Vershinin M. Xu J. Gross S. P. (2008). Stepping, strain gating, and an unexpected force-velocity curve for multiple-motor-based transport. Curr. Biol. 18, 1173–1183. 10.1016/j.cub.2008.07.027 18701289 PMC3385514

[B21] Leduc C. Campas O. Zeldovich K. B. Roux A. Jolimaitre P. Bourel-Bonnet L. (2004). Cooperative extraction of membrane nanotubes by molecular motors. Proc. Natl. Acad. Sci. U. S. A. 101, 17096–17101. 10.1073/pnas.0406598101 15569933 PMC535380

[B22] Leidel C. Longoria R. A. Gutierrez F. M. Shubeita G. T. (2012). Measuring molecular motor forces *in vivo*: implications for tug-of-war models of bidirectional transport. Biophys. J. 103, 492–500. 10.1016/j.bpj.2012.06.038 22947865 PMC3414874

[B23] Li Q. Ferrare J. T. Silver J. Wilson J. O. Arteaga-Castaneda L. Qiu W. (2023). Cholesterol in the cargo membrane amplifies tau inhibition of kinesin-1-based transport. Proc. Natl. Acad. Sci. U. S. A. 120, e2212507120. 10.1073/pnas.2212507120 36626558 PMC9934065

[B24] Mallik R. Petrov D. Lex S. A. King S. J. Gross S. P. (2005). Building complexity: an *in vitro* study of cytoplasmic dynein with *in vivo* implications. Curr. Biol. 15, 2075–2085. 10.1016/j.cub.2005.10.039 16332532

[B25] Martinez J. E. Vershinin M. D. Shubeita G. T. Gross S. P. (2007). On the use of *in vivo* cargo velocity as a biophysical marker. Biochem. Biophys. Res. Commun. 353, 835–840. 10.1016/j.bbrc.2006.12.120 17196170 PMC2889695

[B26] Mclaughlin R. T. Diehl M. R. Kolomeisky A. B. (2016). Collective dynamics of processive cytoskeletal motors. Soft Matter 12, 14–21. 10.1039/c5sm01609f 26444155 PMC4684438

[B27] Milic B. Andreasson J. O. L. Hancock W. O. Block S. M. (2014). Kinesin processivity is gated by phosphate release. Proc. Natl. Acad. Sci. U. S. A. 111, 14136–14140. 10.1073/pnas.1410943111 25197045 PMC4191751

[B28] Nettesheim G. Nabti I. Murade C. Jaffe G. King S. Shubeita G. (2020). Macromolecular crowding acts as a physical regulator of intracellular transport. Nat. Phys. 16, 1144–1151. 10.1038/s41567-020-0957-y

[B29] Ohashi K. G. Han L. Mentley B. Wang J. Fricks J. Hancock W. O. (2019). Load-dependent detachment kinetics plays a key role in bidirectional cargo transport by kinesin and dynein. Traffic 20, 284–294. 10.1111/tra.12639 30809891 PMC6420372

[B30] Rai A. K. Rai A. Ramaiya A. J. Jha R. Mallik R. (2013). Molecular adaptations allow dynein to generate large collective forces inside cells. Cell 152, 172–182. 10.1016/j.cell.2012.11.044 23332753

[B31] Shubeita G. T. Gross S. P. (2012). “Intracellular transport: relating single-molecule properties to *in vivo* function,” in Comprehensive biophysics. Editor Egelman E. H. 1 ed (Elsevier).

[B32] Shubeita G. T. Tran S. L. Xu J. Vershinin M. Cermelli S. Cotton S. L. (2008). Consequences of motor copy number on the intracellular transport of kinesin-1-driven lipid droplets. Cell 135, 1098–1107. 10.1016/j.cell.2008.10.021 19070579 PMC2768369

[B33] Svoboda K. Block S. M. (1994). Force and velocity measured for single kinesin molecules. Cell 77, 773–784. 10.1016/0092-8674(94)90060-4 8205624

[B34] Svoboda K. Schmidt C. F. Schnapp B. J. Block S. M. (1993). Direct observation of kinesin stepping by optical trapping interferometry. Nature 365, 721–727. 10.1038/365721a0 8413650

[B35] Tjioe M. Shukla S. Vaidya R. Troitskaia A. Bookwalter C. S. Trybus K. M. (2019). Multiple kinesins induce tension for smooth cargo transport. Elife 8, e50974. 10.7554/eLife.50974 31670658 PMC6904222

[B36] Vershinin M. Carter B. C. Razafsky D. S. King S. J. Gross S. P. (2007). Multiple-motor based transport and its regulation by Tau. Proc. Natl. Acad. Sci. U. S. A. 104, 87–92. 10.1073/pnas.0607919104 17190808 PMC1765483

[B37] Welte M. A. Gross S. P. Postner M. Block S. M. Wieschaus E. F. (1998). Developmental regulation of vesicle transport in Drosophila embryos: forces and kinetics. Cell 92, 547–557. 10.1016/s0092-8674(00)80947-2 9491895

[B38] Wilson J. O. Quint D. A. Gopinathan A. Xu J. (2019). Cargo diffusion shortens single-kinesin runs at low viscous drag. Sci. Rep. 9, 4104. 10.1038/s41598-019-40550-5 30858425 PMC6411862

[B39] Wilson J. O. Zaragoza A. D. Xu J. (2021). Tuning ensemble-averaged cargo run length via fractional change in mean kinesin number. Phys. Biol. 18, 046004. 10.1088/1478-3975/abf5b3 PMC8460125 33827070

[B40] Xu J. Shu Z. King S. J. Gross S. P. (2012). Tuning multiple motor travel via single motor velocity. Traffic 13, 1198–1205. 10.1111/j.1600-0854.2012.01385.x 22672518 PMC3418377

[B41] Yadav S. Kunwar A. (2022). Sliding of motor tails on cargo surface due to drift and diffusion affects their team arrangement and collective transport. Phys. Biol. 20, 016002. 10.1088/1478-3975/ac99b2 36223776

